# wavess 1.2: Presenting an HLA-aware within-host virus sequence simulation framework

**DOI:** 10.64898/2026.02.19.706869

**Published:** 2026-02-20

**Authors:** Zena Lapp, Thomas Leitner

**Affiliations:** 1 Theoretical Biology and Biophysics, Los Alamos National Laboratory, New Mexico, United States of America

**Keywords:** molecular evolution, immunology, recombination, simulation, virology

## Abstract

**Motivation:**

Understanding how virus sequences are shaped by selection can inform vaccine design and transmission inference. Modeling within-host evolution to interrogate these questions requires a detailed mechanistic framework that accurately captures sequence diversification. The CD8^+^ cytotoxic T-lymphocyte (CTL) response plays an important role in immune-mediated selection and can leave strong signatures in virus sequences; however, existing sequence-based within-host virus modeling frameworks do not explicitly include an HLA-aware CTL response.

**Results:**

We extended our previously published within-host sequence evolution simulator, wavess, to include an explicit CTL response, and share a method for identifying HLA-specific CTL epitopes given a founder virus sequence. We also updated the model to permit a variable recombination rate, which allows for modeling recombination hotspots, non-adjacent genes, and segmented genomes. These extensions to wavess allow for more accurate simulation of viruses and virus genes, particularly in regions of the genome where the immune response is dominated by CTLs (rather than antibodies). It also provides the foundation for investigations of how these newly-added biological mechanisms influence within-host evolution.

**Availability and implementation:**

The core of wavess is written in Python 3, with helper functions written in R. It is available at https://github.com/MolEvolEpid/wavess.

## Introduction

Forward-in-time simulation of virus sequence evolution in a host can provide fundamental knowledge relevant to public health. It can help tease apart how different selective pressures influence virus survival, which may inform vaccine development. It can also improve our understanding of how to correctly infer transmission networks from sequences under strong selective pressures, which may inform inference tools used to suggest interventions. To investigate these questions, the model must accurately simulate mutational signatures in virus sequences.

An important selective pressure that shapes the virus fitness landscape, and therefore sequence signatures, is the CD8^+^ cytotoxic T-lymphocyte (CTL) immune response (Carlson et al., 2015). CTLs recognize non-self peptides presented by human leukocyte antigen (HLA) class I molecules and mount an immune response against infected cells. There is strong pressure on the virus to escape this response, which it can do by mutating such that the peptide can no longer be presented by the host’s HLA molecules. This escape signature can be identified in virus sequence data from infected individuals. However, sequence-based within-host simulators (Haller and Messer, 2023; Jariani et al., 2019) do not explicitly model a CTL immune response that can vary depending on the host’s HLA molecules.

We previously developed a model and R package, wavess (Sambaturu et al., 2025), that simulates within-host virus evolution, with optional recombination and latency, under various selective pressures including conserved sites, replicative fitness, and a generic adaptive immune response with cross-reactivity. Here, we describe two updates to this model, i) an explicit CTL immune response, and ii) variable recombination rates to allow for modeling of recombination hostspots and non-adjacent or segmented sequences. These features are included in wavess version 1.2.

## New developments to wavess

wavess is an individual-based forward-simulation model that explicitly simulates virus sequences including mutation, recombination, and selection (Sambaturu et al., 2025).

### HLA-specific CTL immune response

wavess 1.0 modeled a generic adaptive immune response with cross-reactivity and no option for complete escape; however, the CTL response leads to distinctly observable escape mutations, particularly early in infection (Fischer et al., 2010). As immune-driven evolution in many virus genes is mostly due to the host CTL response rather than the antibody response, we modified wavess to separately model these two components of the immune system. The B-cell antibody immune response is modeled identically to the original wavess immune response. In addition, we added functionality to model complete escape from a CTL response at user-defined amino acid positions. One amino acid is recognized by the immune system; mutation to any other amino acid confers complete and immediate escape from recognition. All T-cell epitopes have an identical user-defined maximum fitness cost *c_max_* (default = 0.5), and each T-cell epitope *i* has a(n optionally) distinct time to reach maximum fitness cost $t_{\max}^{i}$, so T-cell responses can mature at different rates. All responses mature linearly from 0 to $t_{\max}^{i}$ and once $t_{\max}^{i}$ is reached, that cost is maintained for the remainder of the infection. So the fitness of a recognized epitope *i* in generation *t* is defined as:

(1) $$F_{I_{T}}^{i}\left(t\right)=1-c_{\max}\times \min\left(t/t_{\max}^{i},1\right)$$

Epitopes that have escaped recognition have a fitness of 1. The T-cell immune fitness for a virus at generation *t* is the product of the individual fitness for each epitope:

(2) $$F_{I_{T}}\left(t\right)=\overset{m}{\underset{i=1}{\prod }}F_{I_{T}}^{i}\left(t\right)$$

where *m* is the total number of CTL epitopes in the individual.

The overall fitness for a virus at time *t* is the product of the virus’s conserved fitness *F_C_*, replicative fitness *F_R_*, B-cell (antibody) fitness *F_I_B__*, and T-cell (CTL) fitness *F_I_T__*:

(3) $$F\left(t\right)=F_{C}\times F_{R}\times F_{I_{B}}\times F_{I_{T}}$$

### Variable recombination rate

wavess 1.0 used an identical recombination rate (a combined rate of dual infection followed by recombination) across all potential breakpoints. To enable modeling of recombination hotspots, recombination between non-adjacent genes in a sequence, and reassortment of genes on separate segments of segmented viruses, we implemented the option to have a variable recombination rate across the simulated sequence.

Within wavess, we convert the recombination rate into a probability of recombination by assuming that the number of recombination events follows a Poisson process. In wavess 1.0, we assumed that the recombination rate was small enough that, per generation, only zero or one recombination events would occur at any given breakpoint; however, larger recombination rates break this assumption. Therefore, we now calculate the probability of an odd number of recombination events occurring as (1 – *e*^−2*n*λ^)/2, because if an even number occurs then it is not observable in the sequence. Rates over 3 recombinations per breakpoint per generation lead to a probability of about 0.5.

As identifying recombination events at each breakpoint individually substantially increases runtime, we implemented an algorithm that determines, based on the recombination probabilities, the optimal method to identify recombination events across the sequences. When the majority of positions (> 95%) have the same baseline rate, binomial sampling is used to identify recombination events at those positions. For positions that differ from the baseline, or if no baseline exists, recombination events are identified at each position individually. We used GitHub Co-Pilot to help with optimization.

We envision that variable recombination rates can be used to model i) recombination hotspots, ii) non-adjacent genes, with a higher recombination rate between the genes, and iii) segmented genomes where the rate indicates the reassortment rate. For the second scenario, the rate can be calculated as *n*λ where *n* is the number of breakpoints between the genes and λ is the baseline recombination rate for a single breakpoint.

### Usage updates and availability

We have updated the wavess R package vignettes to describe, and provide examples of, the new options for a CTL immune response and a variable recombination rate. All updates can be found at https://github.com/MolEvolEpid/wavess.

## HIV-1 example with an HLA-aware framework

### Input data and simulation settings

To test our model, we simulated within-host HIV-1 evolution of *pol* and *gp120* (spliced together) using the wavess default values unless otherwise noted. *pol*, the polymerase gene, is under strong CTL immune pressure but not strong antibody pressure, while *gp120*, an envelope protein, is heavily targeted by the host antibody response. We used as the founder sequence *pol* and *gp120* from subtype B sequence DEMB11US006 (GenBank accession number KC473833) (Sanchez et al., 2014). Conserved sites were identified as recommended in wavess for *pol* and *gp120* and mapped to the founder sequence. The LANL HIV database consensus sequence (Linchangco et al., 2022) was used as the reference for both genes. B-cell epitope locations were identified for *gp120* only, using the recommended method from wavess with the LANL HIV database ENV sequence features. The same 10 B-cell epitopes were used for all simulations.

### CTL epitopes

**Figure 1:**
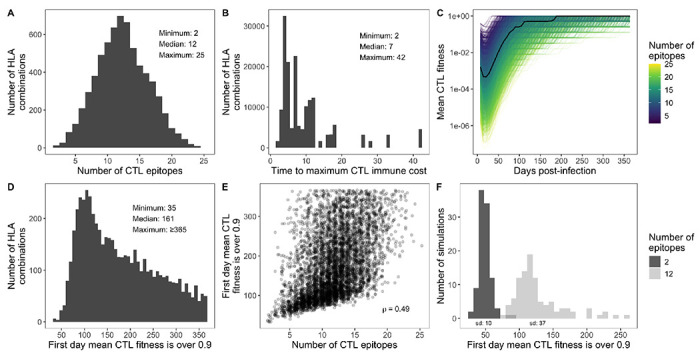
Escape from the CTL response. (A) Number of CTL epitopes across all combinations of two HLA-A and HLA-B molecules from the IEDB 27 CTL panel. (B) Distribution of time to maximum CTL immune response for all epitopes from panel A. (C) Mean CTL fitness output by wavess for each simulation. (D) First day the mean CTL fitness was > 0.9 for each simulation. (E) Correlation between the number of CTL epitopes and the first day the mean CTL fitness was > 0.9. *ρ* is Spearman’s. (F) First day the mean virus CTL fitness was > 0.9 for each of 100 replicates of two HLA combinations with different numbers of epitopes. The standard deviation is indicated under each histogram.

We identified founder T-cell epitopes for the IEDB panel of 27 HLA-A and HLA-B alleles (at the subtype, i.e. 4-digit, level) using the IEDB T-cell prediction tool with the NetMHCpan 4.1 EL prediction method for MHC-1 Binding/Elution (Reynisson et al., 2020; Vita et al., 2025). We also calculated class I pMHC immunogenicity with 1,2,C terminals masked. We calibrated the epitope filtering criteria to align with values in the literature, while allowing different sequences and HLAs to lead to different numbers of recognized epitopes. We wanted a filtering criteria that did not lead to zero CTL epitopes, but resulted in few enough to be realistic. It is estimated that between 9-18 escape mutations become fixed in the population within six months of infection (Carlson et al., 2015), with the epitope-specific time to maximum T-cell response ranging from less than two weeks to over two months (Turnbull et al., 2009). With this in mind, for any given set of 2 HLA-A and 2 HLA-B alleles, we identified all epitopes of length 9 amino acids under a NetMHCpan percentile of 0.1 and with an immunogenicity score of less than 1/90. Across all HLA combinations, this resulted in a median of 12 epitopes across *pol* and *gp120* (range 2-25; Figure 1A). The number of days to reach maximum CTL immune cost was considered to be the inverse of the epitope immunogenicity score. Across all epitopes, the median time was 7 days (range 2-42; Figure 1B). For each epitope, the anchor positions (2 and 9) were considered positions at which a mutation would lead to escape from the CTL response (Carlson et al., 2015).

### Recombination rate

We set the baseline recombination rate within each gene to *r* = 1.5 × 10^−5^ per breakpoint per generation, and the rate between the two genes to *r* × *n*, where *n* = 1128, the number of nucleotides between *pol* and *gp120* in the founder, yielding a rate of 0.17.

To investigate signals of recombination breakpoints in the simulated sequences, we used 3seq (Lam et al., 2018). To minimize compute time, we identified breakpoints using sequences sampled from the last 10 weeks of the simulation (*n* = 200 sequences) for 10 randomly selected simulations.

### Simulations

For each combination of 2 HLA-A and 2 HLA-B epitopes (*n* = 6600), we simulated within-host evolution using wavess for one year. Weekly, we sampled 20 sequences and recorded the mean CTL fitness. For the HLA combination with the smallest number of epitopes (A*23:01, A*01:01, B*40:01, B*08:01) and one with a median number of epitopes (A*02:01, A*01:01, B*15:01, B*57:01) we also performed 100x replicates to investigate the stochasticity of CTL fitness over time. As complete immune escape may or may not occur at conserved sites (Li et al., 2007; Peyerl et al., 2004), we set the conserved cost and the maximum T-cell immune cost to 0.5.

### Model output exploration and analysis

#### CTL response

Different HLA combinations led to different days at which the mean CTL fitness was > 0.9 (median 161, range 35 to ≥ 365; Figure 1C–D), which was correlated with the number of epitopes recognized by the CTL response (Spearman *ρ* = 0.49; Figure 1E). This also aligns with reports of escape occurring between a few weeks to several years post-infection (Goonetilleke et al., 2009). Furthermore, the time to escape for viruses with fewer recognized epitopes was less variable than the time to escape for viruses with more recognized epitopes (standard deviation of 10 days for 2 epitopes vs. 37 days for 12 epitopes; Figure 1F).

#### Recombination breakpoints

**Figure 2:**
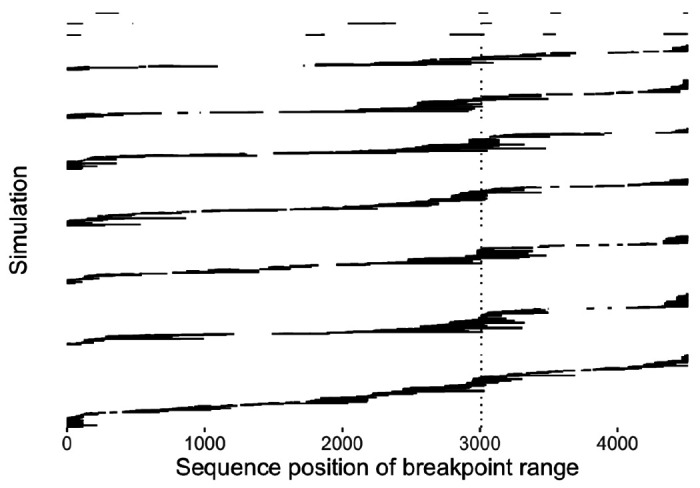
Example recombination breakpoints. Recombination breakpoints identified by 3seq for 10 simulations. 3seq returns a range for each recombination breakpoint; this is what is depicted in each row. The vertical dotted line indicates where *pol* and *gp120* were spliced; to the left is *pol* and to the right is *gp120*.

As expected, breakpoints were often identified at location where *pol* and *gp120* were spliced in the simulated sequence, although recombination events were detected throughout the sequence (Figure 2).

## Conclusion

wavess can now model non-adjacent or segmented sequences, variable recombination, latency, conserved sites fitness, replicative fitness, antibody fitness, and CTL fitness. The new features we added here allow for more accurate within-host modeling of virus genes under CTL-mediated selective pressures. These updates may also prove useful for modeling non-virus pathogens.

## Data Availability

The data and code in this article are available at https://github.com/MolEvolEpid/wavess_ctl_manuscript.
